# Construction of Topological Bound States in the Continuum Via Subsymmetry

**DOI:** 10.1021/acsphotonics.4c00600

**Published:** 2024-07-17

**Authors:** Xiangdong Wang, Domenico Bongiovanni, Ziteng Wang, Amgad Abdrabou, Zhichan Hu, Dario Jukić, Daohong Song, Roberto Morandotti, Ramy El-Ganainy, Zhigang Chen, Hrvoje Buljan

**Affiliations:** † TEDA Applied Physics Institute and School of Physics, Nankai University, Tianjin 300457, China; ‡ INRS-EMT, 1650 Blvd. Lionel-Boulet, Varennes, Quebec J3X 1S2, Canada; § Elmore Family School of Electrical and Computer Engineering, Purdue University, West Lafayette, Indiana 47907, United States; ∥ Faculty of Civil Engineering, University of Zagreb, A. Kačića Miošića 26, Zagreb 10000, Croatia; ⊥ Collaborative Innovation Center of Extreme Optics, Shanxi University, Taiyuan, Shanxi 030006, China; # Department of Physics, Michigan Technological University, Houghton, Michigan 49931, United States; ¶ Department of Physics, Faculty of Science, University of Zagreb, Bijenička c. 32, Zagreb 10000, Croatia

**Keywords:** bound states in the continuum, subsymmetry, topological photonics, topological phase of matter, photonic lattices

## Abstract

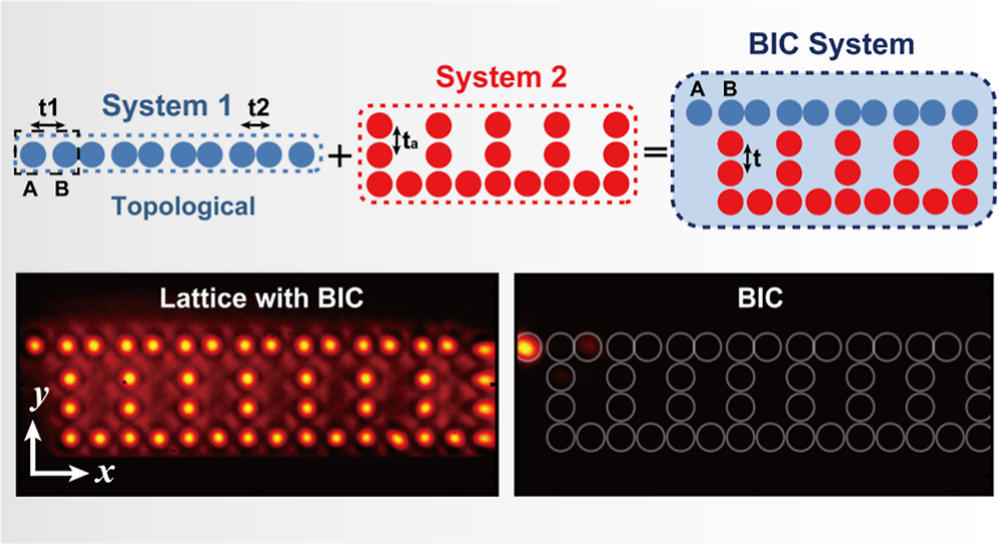

Topological bound states in the continuum (BICs) are localized topological boundary modes coexisting with a continuous spectrum of extended modes. They have been realized in systems with symmetry-protected topological phases, where their immunity to defects and perturbations depends on the presence of symmetries. Here we propose a method that transforms an in-gap topological boundary state into a BIC by using the concept of subsymmetry. We design the coupling between a system possessing in-gap topological modes and a system possessing a continuum of states that results in topological BICs. We define the criteria for the coupling that yields the desired results. To implement this scheme, we construct representative topological BICs based on one-dimensional Su–Schrieffer–Heeger models and implement them in photonic lattices. Our results not only reveal novel physical phenomena but may also provide methods for designing a new generation of topological devices.

## Introduction

Bound states in the continuum (BICs) are extraordinary wave phenomena^1−3^ with the potential for creating ultrahigh-Q devices.^2,3^ They are ubiquitously present in both natural and artificial systems,^4−9^ stimulating substantial interest in both theory and experiment.^10−23^ BICs appear predominantly because of destructive interference at some specific frequencies and orientations, thereby engendering orthogonality between the localized bound modes and the embedded continuum spectrum.^2,3,24^ Thus, a widespread method to realize BICs in specific structures is predicated on the destructive interference enforced by geometry and symmetry.^25,26^ However, unavoidably, imperfections in realistically fabricated samples can readily disrupt these stringent geometric prerequisites and the other conditions for the manifestation of BICs. Therefore, there is motivation for exploring new schemes for creating (topological) BICs, which do not require, or at least have less restrictive requirements, for the presence of symmetries.

Of particular interest are the topological BICs with edge and corner states in topological insulators (TIs).^27−31^ The boundary states of TIs are hallmarked by their distinct localization and remarkable robustness to perturbations.^32,33^ In this context, topological BICs, which enjoy protection due to band topology, would be an appealing choice for practical applications. However, the conventional bulk-edge correspondence determines that boundary states are typically located within the band gap, thereby presenting a fundamental contradiction with the BIC concept. Based on that, only a limited number of topological models, including higher-order topological phases, can support BICs.^34−40^ Notably, some of these models necessitate the presence of additional crystalline symmetry, akin to topological crystalline insulators,^41^ to ensure that the boundary states persist as BICs rather than devolving into standard resonances.

Here, we propose a simple yet general method that transforms an in-gap topological boundary state into a BIC. The method is based on the coupling of a TI possessing an in-gap zero-energy boundary mode to a system with a continuum of modes that encompasses zero-energy. When the coupling is negligible, the boundary state becomes a BIC (this is somewhat trivial because subspaces are separated). However, inspired by the recently proposed subsymmetry (SubSy) concept,^42^ we demonstrate analytically that a BIC can maintain its localized nature and topological protection even when the coupling is strong if it adheres to the specific criteria defined below. Both theoretical and experimental validations of this proposal are accomplished by constructing a BIC with a topological subsystem being a one-dimensional (1D) Su–Schrieffer–Heeger (SSH) lattice or a plurality of 1D SSH lattices. Namely, it is feasible to extend this idea to many judiciously coupled subsystems, with a large number and variety of BICs. Specifically, we experimentally detect BICs in a triply-stacked 1D SSH and an armchair-like lattice configuration. Our method offers a versatile path for generating topological BICs with adjustable features, which may have potential applications in the development of novel photonic devices.

## Results

**Figure 1:**
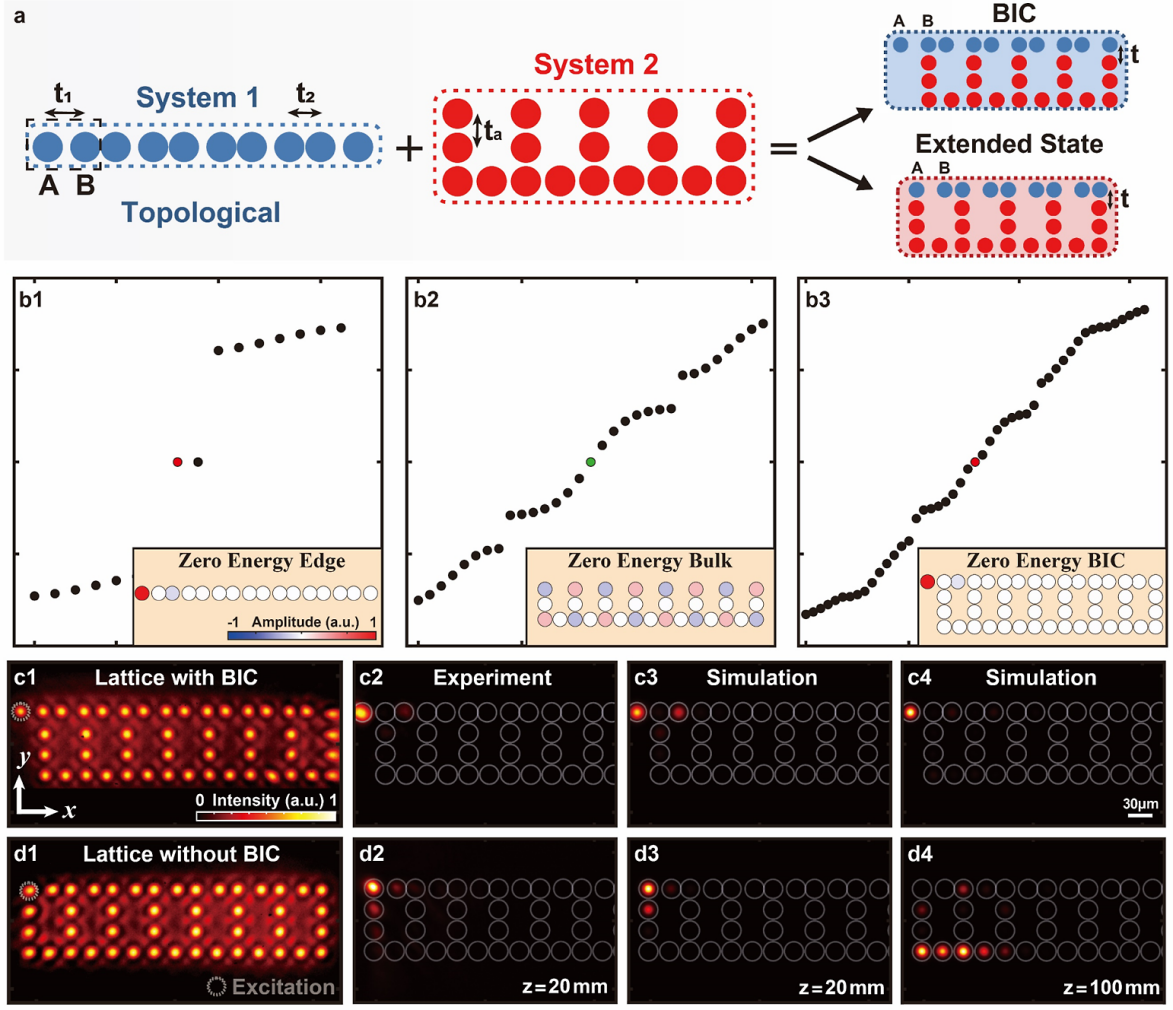
Illustration of a method for constructing a global system with a single BIC mode. (a) Sketch of a topologically nontrivial 1D SSH model (System 1) with a black-dashed square indicating the unit cell, a uniform armchair-like model (System 2), and two ways of coupling the two systems; the global system in the upper right panel possesses a BIC, whereas the one in lower panel does not. (b) Spectrum (band structures) calculated for System 1 (b1), System 2 (b2), and the global system possesing a BIC (b3). Propagation constants are shown on the *y*-axis with the same scale in each panel of (b1-b3), whereas the mode numbers are on the *x*-axis. The inset in each panel of (b1-b3) displays the amplitude distribution of the zero-energy eigenmode. (c1,d1) Experimental photonic lattices representing the two global systems established by the point-to-point laser-writing technique, designed to satisfy (c1) or violate (d1) Condition 1. (c2,d2) Corresponding output intensities after 20 mm-long propagation; initially, one single waveguide is excited at the uppermost-left A site. The light in (c2) remains exponentially localized within A sublattice sites, which is indicative of topological nature, while that in (d2) spreads across multiple sublattices, especially in the neighboring B site, indicating the absence of the topological edge state. (c3–d4) Numerical simulation corresponding to the experiments in (c2,d2) after 20 mm (c3,d3) and 100 mm (c4,d4) of propagation, showing dramatic difference in the two lattices.

We start our theoretical analysis by considering a global system formed by two coupled subsystems. As shown in the schematic illustration of Figure 1a, the topological phase of the first subsystem (referred to as System 1) is nontrivial (i.e., it is a TI), while the topological status of the second subsystem (referred to as System 2) is not defined *a priori*. This freedom provides an opportunity to design a system featuring BIC and topological properties. The Hamiltonian *H* associated with the global system can be written as
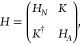
1where *H*_*N*_ and *H*_*A*_ are the two uncoupled sub-Hamiltonians describing Systems 1 and 2, respectively, and *K* encodes their coupling. The eigenvalues *E* and eigenmodes ψ of *H* can be found by solving the eigenvalue problem *H*ψ = *E*ψ. Likewise, we can obtain the eigenvalues *E*_*N*_ and *E*_*A*_ and eigenmodes ψ_*N*_ and ψ_*A*_ associated with the two independent subsystems. When there is no coupling between the two subsystems (*K* = 0), the two vectors [ψ_*N*_, 0]^*T*^ and [0, ψ_*A*_]^*T*^ are still eigenmodes of the global system. In this case, a topological boundary mode (denoted with ψ_*N*0_), which is supported by the nontrivial System 1, gives rise to the boundary eigenmode of the global Hamiltonian *H*: ψ_0_ = [ψ_*N*0_,0]^*T*^.

If the two subsystems are coupled, i.e., *K* ≠ 0, the wave function ψ_0_ is not necessarily an eigenstate of *H*. However, if *K* satisfies the following relation
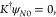
2then the boundary mode ψ_0_ remains an eigenstate of *H* with the same eigenvalue *E*_*N*0_. The condition introduced in eq 2 is referred to as Condition 1. The boundary modes are manifested in finite-sized systems for which the Hamiltonian is most conveniently written in real space. Therefore, in specific examples presented below, Condition 1 is tested in the real space representation of *H*.

Topological boundary modes are usually located in an energy gap of a topological system. To construct a boundary mode ψ_0_ of *H*, which is at the same time the BIC mode, System 2 should provide a continuum spectrum encompassing the eigenvalue *E*_*N*0_ of ψ_0_. Since Condition 1 does not enforce any restrictions on the characteristics of System 2, we can always find System 2 with such a spectrum. However, we must ensure that the gap will not open at the eigenvalue *E*_*N*0_ due to the coupling of the two systems. It seems plausible that if we weakly couple System 1 and System 2, this gap will not open. A more rigorous condition can be derived as follows. If we consider System 1, System 2, and the global system *H* to be lattices (which is often the case in photonic systems), their band gap structure is calculated by expressing the Hamiltonian *H* in the momentum space (i.e., *k*-space) representation, that is, for an infinite lattice with an appropriately chosen unit cell. The eigenvalue *E*_*N*0_ overlaps with a continuous band if there is a solution for ψ′ to satisfy the following equation (referred to as Condition 2) in momentum space
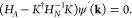
3

In eq 3, we have set the eigenvalue *E*_*N*0_ to be zero without loss of generality. In summary, if both Conditions 1 and 2 are satisfied, then ψ_0_ is a topological BIC. Its topological protection is inherited from System 1. Condition 1 is checked in the real space, whereas Condition 2 is checked in momentum space.

To provide an illustrative example, for System 1 we choose perhaps the simplest topologically nontrivial 1D lattice, i.e., the SSH model with A and B sublattices (see Figure 1a). The SSH model was initially proposed to describe the polyacetylene chain.^43,44^ Since then, it has been realized in a plethora of versatile platforms, such as photonics and nanophotonics,^45−48^ plasmonics^49^ and quantum optics,^50^ and also in the context of parity–time symmetry and nonlinear non-Hermitian phenomena.^51,52^ The topological phase of a 1D SSH lattice is determined by the ratio between intra- and intercell hopping amplitudes, denoted as *t*_1_ and *t*_2_, respectively. As shown in Figure 1b1, the 1D SSH lattice exhibits nontrivial topological properties with in-gap edge states pinned at zero energy when *t*_1_ < *t*_2_. The distribution of zero-energy edge states possesses chirality, which means the left edge state only distributes in the A sublattice while the right edge state resides only in the B sublattice.

Recently, it has been shown that for the robustness of the edge modes in 1D SSH lattices, the so-called SubSy topological protection is sufficient.^42^ In the SSH model, SubSy means that the operator equation defining the chiral symmetry holds only on one sublattice, thus, there is A-SubSy where the chiral symmetry equation holds only on the A sublattice, and equivalently for the B-SubSy.^42^ Without losing generality, we consider the left-edge mode residing on the A sublattice for constructing BICs. With any perturbation of the SSH model satisfying the A-SubSy, this left edge state will remain localized only on the A sublattice with a zero-energy eigenvalue. Thus, if the coupling between System 1 (the SSH lattice) and System 2 is implemented only through the B sublattice sites of the SSH model, then Condition 1 [i.e., eq 2] will be satisfied as the left edge mode is located on the A sublattice. In other words, ψ_0_ will always be the eigenmode of the global system as long as *K* has no coupling to the A sublattice, although it can contain coupling to the B sublattice sites.

For ψ_0_ to be a BIC, the global system *H* also needs to provide a bulk band encompassing the zero-energy, i.e., Condition 2 must be satisfied. There are various candidates for System 2 to satisfy Condition 2. One possible example is an armchair-like lattice with only one coupling parameter *t*_*a*_ illustrated in Figure 1a. The band structure and bulk–mode profile at zero energy of this System 2 are illustrated in Figure 1b2.

Figure 1a illustrates two different constructions of the global system. One configuration (upper right panel) satisfies both Conditions 1 and 2, and therefore supports a topological BIC. The other one (lower right panel) does not respect Condition 1 because System 1 and System 2 are coupled through the A sublattice sites; it supports a zero-energy resonance mode. The band structure and the mode profile ψ_0_ of the global system from the upper inset of Figure 1a are shown in Figure 1b3. In this calculation, the coupling parameters indicated in Figure 1a,b1–b3 are *t*_1_ = 0.2, *t*_2_ = 2, *t*_*a*_ = 1 and *t* = 1.

To experimentally demonstrate the existence or absence of the BIC boundary modes in the global system, two different sets of photonic waveguide arrays (Figure 1c1,d1) are established by employing continuous-wave laser writing technique within a 20 cm-long Strontium Barium Niobate (SBN:61) photorefractive crystal.^37,52^ The lattice spacings are 26 and 34 μm in a 1D SSH lattice (System 1), and 30 μm in an armchair-like lattice (System 2), corresponding to the coupling conditions in the tight-binding model. The writing process is carried out by applying an electric field of 150 kV/m along the *c*-axis of the crystal, which enables the translation of light intensity into reconfigurable refractive index changes. The experimental setup includes a spatial light modulator to control the amplitude and phase of the initial Gaussian beam, shaping the probe beam to test the photonic lattices shown in Figure 1c2,d2. To maintain a linear regime throughout propagation, the probe beam is set at a very low power (on the order of nW) to prevent nonlinear self-action. Since the photonic lattices are optically induced by nonlinearity, we employ the nonlinear Schrödinger equation with parameters close to those from experiments to simulate light propagation through the lattices (shown in Figure 1c3,c4,d3,d4).

The probe beam is injected at the top-left corner site in both lattices. This excites dominantly the BIC mode on the left edge, however, some of the bulk modes are inevitably excited as well. This can be seen from the structure of the BIC mode, which exponentially decays from the corner along the upper edge, and is present solely on the A sublattice, as illustrated in the inset of Figure 1b3. The corresponding output intensities after 20 mm of propagation are shown in Figure 1c2,d2. The output intensity (at 20 mm) resides dominantly on the A-sublattice sites for the Condition-1-preserving lattice structure, with a negligible amount of power in the second site which is in the B sublattice. For a direct comparison, the output intensity in the Condition-1-breaking lattice is observed to strongly leak light into the B sublattice, and therefore into System 2 (bulk) already after 20 mm of propagation, implying the absence of the BIC (Figure 1d2). Numerical simulation results after 20 mm of propagation distance agree with the experimental observations (Figure 1c3,d3). Moreover, simulations to a longer propagation distance of 100 mm through the lattice with the Condition-1-preserving condition show that only the BIC mode remains localized in the upper left corner, and the rest of the power dissipates into the bulk (Figure 1c4). In contrast, simulations to 100 mm of propagation under the breaking condition corroborate the absence of the BIC mode (Figure 1d4). Our numerical and experimental results demonstrate that the global system generates a BIC mode when respecting Conditions 1 and 2. On the other hand, in a scenario where Condition 1 is violated, the BIC mode is absent.

**Figure 2:**
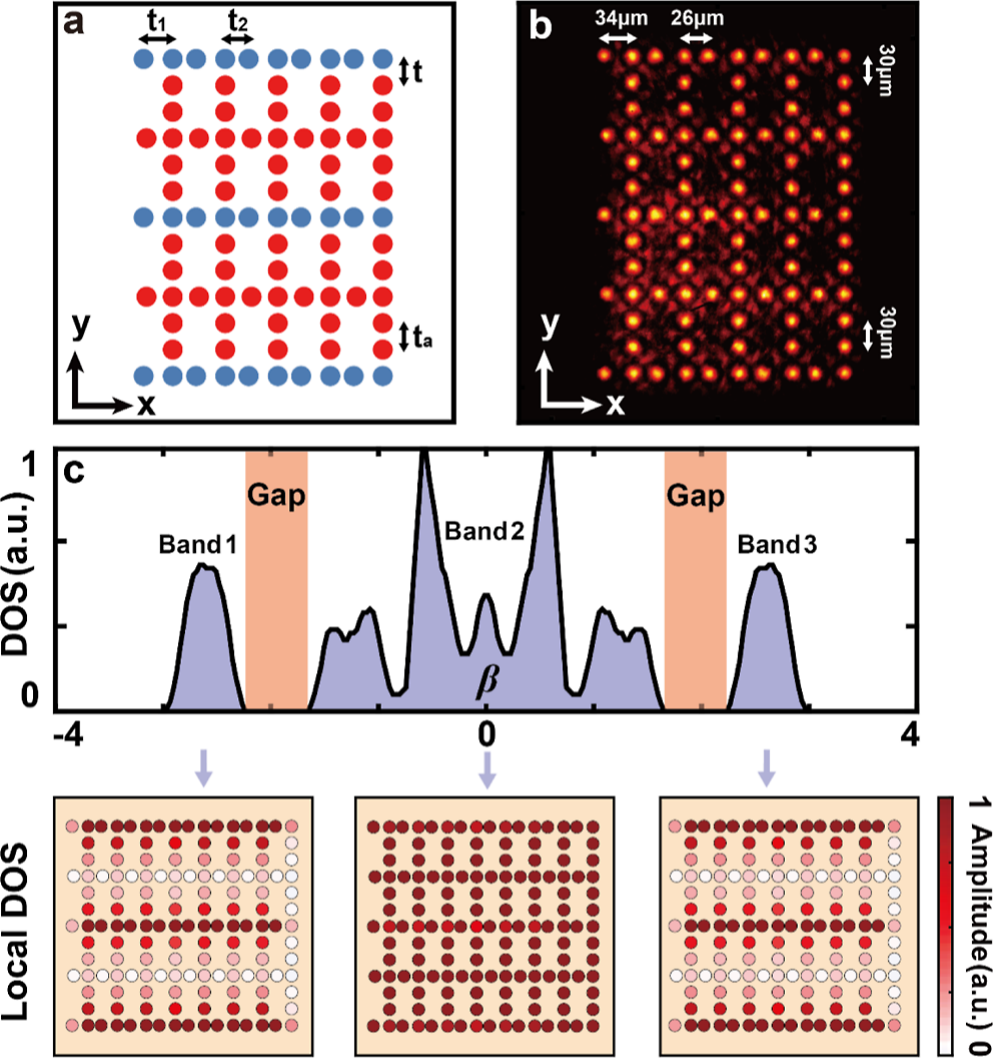
A topological multi-BIC system composed of five interconnected subsystems. (a) Illustration of the lattice structure for a global system consisting of three topologically nontrivial 1D SSH models (blue circles) and two armchair-like lattices (red circles). (b) Corresponding lattice established in experiment, where the lattice spacings corresponding to different coupling amplitudes are indicated by white arrows. (c) DOS (upper panel) reveals the presence of three continuous bulk bands demarcated by two bandgaps. The lower three panels plot the local DOS corresponding to the three bands. The local DOS is calculated by adding absolute values squared of every eigenstate from a given band; for the clarity of presentation, the plots show normalized local DOS in a scale from zero to one. White lattice sites indicate that not a single eigenstate from a given band populates that site.

The discussion above focuses on a global system *H* composed of only two subsystems. However, we may ask whether it would be possible to construct a global system by integrating three or even more subsystems, so to enable the control of an arbitrary number of BIC modes. The answer to this question is yes. To demonstrate this, we consider a complex lattice structure illustrated in Figure 2a, which comprises five subsystems: three subsystems are represented by three topologically nontrivial 1D SSH lattices (top, middle, and bottom; blue circles), and they are interconnected with two topologically trivial subsystems (red circles) with a single coupling parameter *t*; the distance between lattice sites in the trivial systems is half of the size of the unit cell in the SSH models. The coupling parameters indicated in Figure 2a used in the calculation are *t*_1_ = 0.2, *t*_2_ = 2.5, *t* = 0.6, and *t*_*a*_ = 0.6. A corresponding lattice structure established experimentally by the laser-writing technique is shown in Figure 2b. The density of states (DOS) calculated for this complex lattice, plotted in Figure 2c, reveals the existence of three bands and two gaps, with representative local DOS shown at the bottom panels.^35,36^ The local DOS is evaluated at each single site with contributions from all modes in the corresponding band, which varies in amplitude across the lattice.

**Figure 3:**
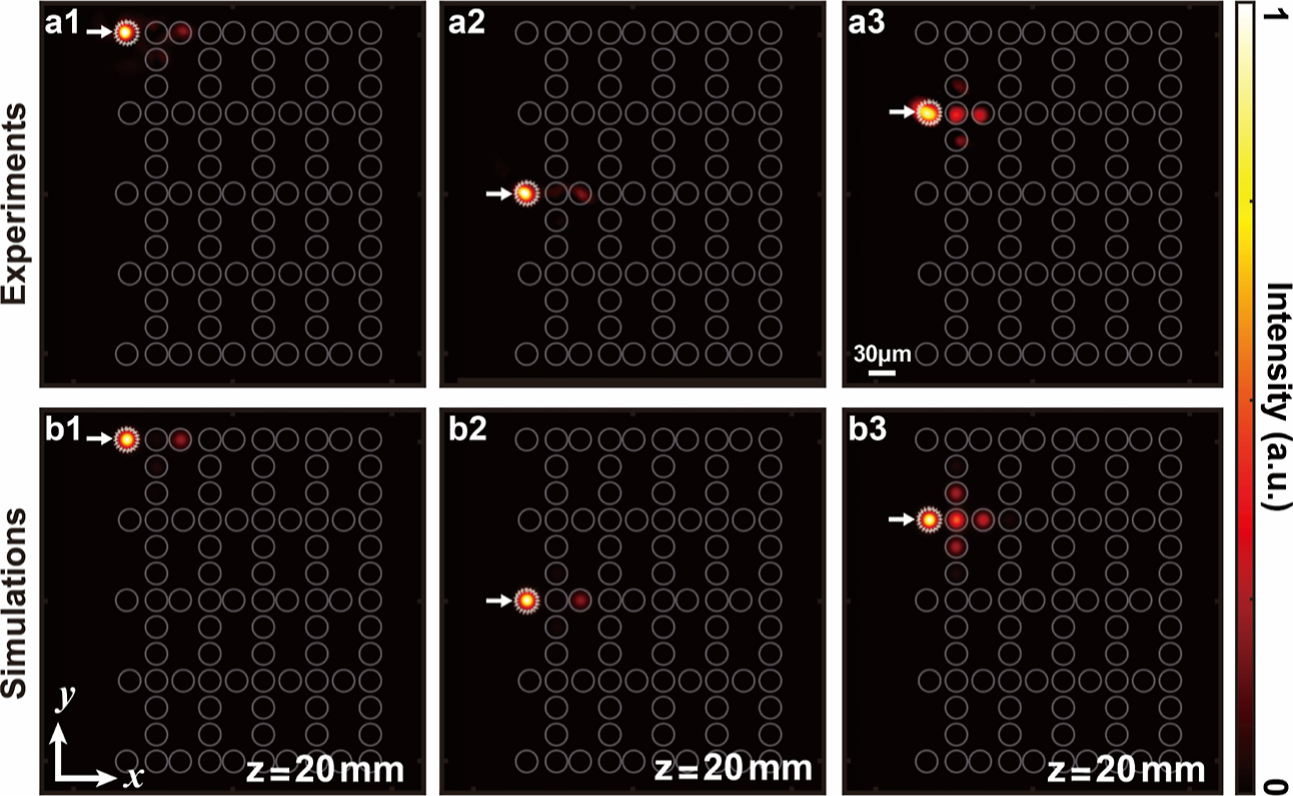
Experimental and simulation results in a multi-BIC system. (a1–a3) Output intensity measurement after 20 mm propagation of a probe beam through the lattice; the probe beam initially excites only a single lattice site indicated with a white arrow in each subplot. Localization of light is observed at the excited lattice site in (a1) and (a2), with a weak distribution in the next-nearest-neighbor lattice site of the System 1, indicating high amplitude of BIC modes at the initially excited lattice sites. For the excitation in (a3), there is an absence of localization as light goes to nearest-neighbor sites and beyond, indicating there is no BIC mode at the lattice site illuminated initially. (b1–b3) Numerical simulations corresponding to the experimental results in (a1–a3), showing good agreement.

Experimental and numerical results are presented in Figure 3a,b, respectively, demonstrating that the system also supports three distinct BIC modes inherited from the three nontrivial 1D SSH lattices at zero energy (embedded in the central band). In Figure 3a1 we excite the upper left corner of the top SSH lattice, which excites dominantly the upper left BIC mode, but somewhat also the bulk modes. Experiments show that after 20 mm of propagation, most of the light is present in the upper left corner and the third waveguide in the upper row, which corroborates the fact that the system supports a topological BIC mode. Simulations at 20 mm of propagation (Figure 3b1) agree with experiments. The lower left BIC mode is equivalent to the upper left one due to the symmetry of the system and therefore we omit discussing it. The experimental parameters for Figure 3 are the same as those used in Figure 1c1,d1.

In Figure 3a2 we input the light in the middle-left edge site of the lattice, which excites dominantly the middle-left BIC mode, but also the bulk modes. Experiments show that after 20 mm of propagation, most of the light is present in this BIC mode. Simulations at 20 mm of propagation (Figure 3b2) agree with experiments.

In contrast, when a lattice site on the left edge that does not correspond to the location of the BIC modes is excited, a significant leakage of the output intensity into the bulk region is observed (Figure 3a3) already after 20 mm of propagation, which is corroborated by the simulations (Figure 3b3). Both numerical simulations and experimental observations validate the applicability of our approach to create systems with multiple topological BICs inherited from a multitude of topological subsystems. To underpin the observations presented in Figure 3, we conduct another set of experiments, where we excite the topologically nontrivial edge mode with its characteristic out-of-phase pattern and compare it with the initial condition where the same waveguides are excited but all in-phase. We observe a localized mode in the first case, but delocalization and light leakage into the B sublattice in the second case (see Figure S1 in the Supporting Information).

Before closing, let us discuss the robustness of this scheme to create the BIC modes. The most general (Hermitian) perturbation on the coupling *K* and System 2 (*H*_*A*_) is
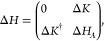
4

We do not address how perturbations of System 1 (*H*_*N*_) affect its topological mode because this question was addressed in a plethora of studies before (e.g., see ref (42) and references therein). The perturbation Δ*H* can be split into two main contributions
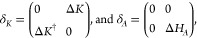
5where δ_*K*_ and δ_*A*_ represent different perturbations with respect to the two conditions. By solving for eigenstates and eigenvalues of the perturbed Hamiltonian *H* + Δ*H*, we perform a robustness analysis of the BIC ψ_0_ = [ψ_*N*0_,0]*^T^*. We emphasize that Condition 1 ensures that the mode ψ_0_ and its eigenvalue *E*_*N*0_ remain unchanged, while Condition 2 guarantees that the bulk band encompasses *E*_*N*0_ (i.e., zero energy).

Regarding the δ_*K*_ perturbation, it has the potential to violate both Condition 1 and 2. For δ_*K*_ that violates Condition 1, ψ_0_ is no longer a BIC. For δ_*K*_ that satisfies Condition 1, i.e., (*K*^†^ + Δ*K*^†^)ψ_*N*0_ = 0, ψ_0_ and *E*_*N*0_ remain unchanged. In the latter case, if δ_*K*_ does not violate Condition 2, ψ_0_ remains to be a BIC; however, if δ_*K*_ violates Condition 2, then ψ_0_ is an in-gap bound state. The δ_*A*_ perturbation has the potential to violate only Condition 2. When it does, ψ_0_ becomes an in-gap bound state but remains robust due to its topological nature. Which perturbations will violate Condition 1 and/or 2 depends on the specific system.

**Figure 4:**
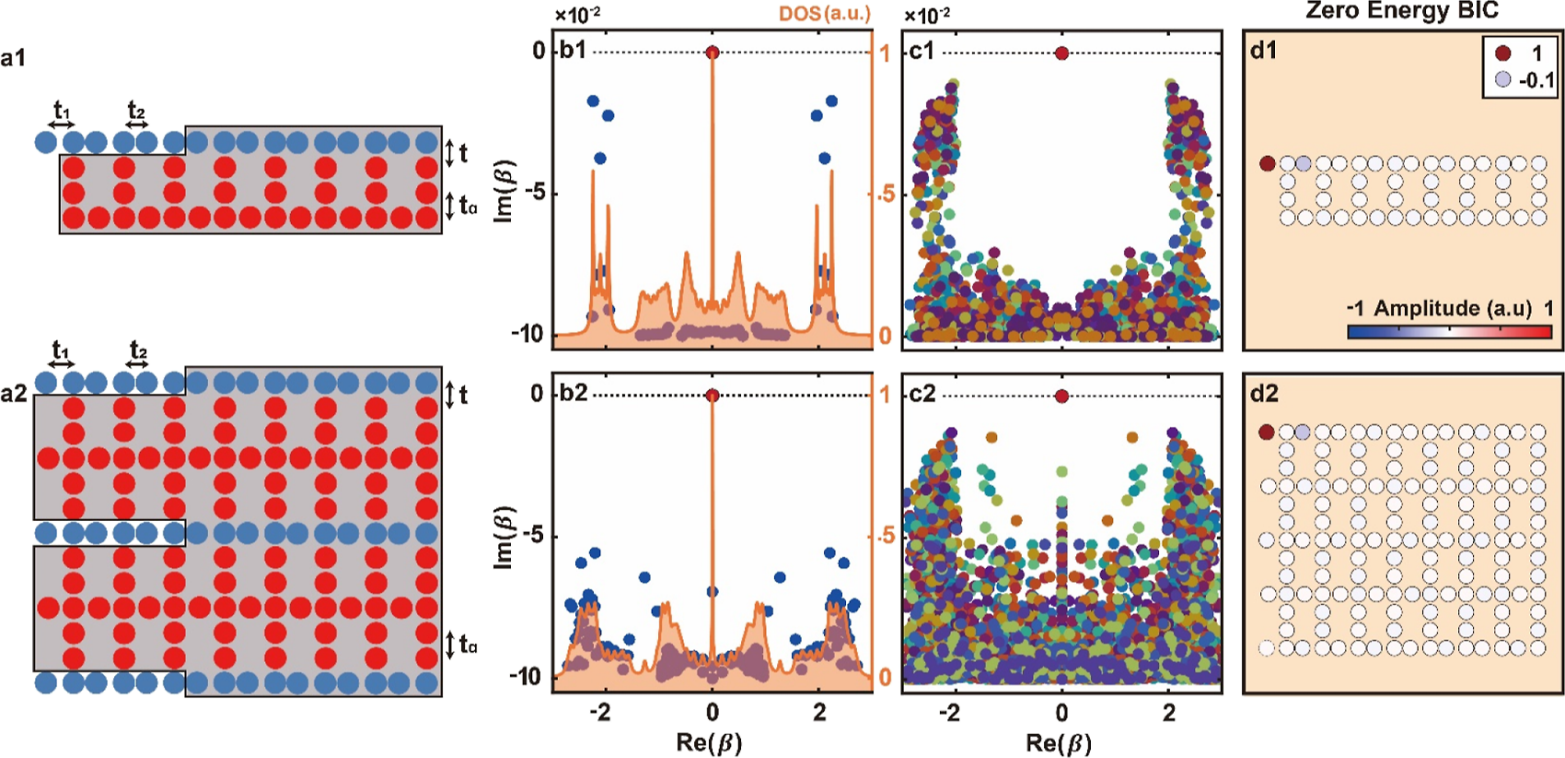
Numerical robustness analysis of the zero-energy BICs. (a1,a2) Schematic illustration of a global system with a single BIC (a1) or multi-BICs (a2), satisfying Conditions 1 and 2. The gray shaded region highlights the “loss mask” with a magnitude of 0.1 introduced in the bulk. System 1 is shown with blue lattice sites, and System 2 with red lattice sites. The coupling between System 1 and System 2 (i.e., *K*) is denoted with *t*. Schematics are not to scale. Results shown in (b1–d1) correspond to the lattice in (a1), while results in (b2–d2) correspond to the lattice in (a2). (b1,b2) The eigenvalues of the unperturbed lattices plotted in the complex plane (blue dots) and a BIC (top red dot) with the presence of the loss mask. The orange shaded region plots the corresponding normalized DOS. (c1,c2) Superimposed eigenvalues calculated for 50 different random perturbations with δ = 0.5; the loss mask is present. Dots with the same color refer to eigenvalues for the same set of perturbations. Both lattice structures display boundary states pinned at the zero energy (marked with red dots at the top) immune to perturbations. (d1,d2) Mode distributions of the boundary zero-energy modes in the two lattices, where all white dots represent zero amplitude. There are three zero-energy modes for the lattice in (a2), but only one is shown. See text for the values of the indicated coupling parameters.

In what follows, we conduct numerical robustness analysis of topological boundary states of the two models mentioned above. We consider lattices shown in Figure 4a1,a2, where *t*_1_ and *t*_2_ are the intracell and intercell hopping parameters of System 1 (same as in Figures 1 and 2; the 1D SSH model is represented with blue lattice sites). System 2 (*H*_*A*_) is shown with red lattice sites; all couplings between red sites are initially set to *t*_*a*_. The couplings between System 1 and 2 (i.e., between red and blue sites corresponding to *K* in the Hamiltonian) are denoted with *t*. Unperturbed hopping parameters are *t*_1_ = 0.2, *t*_2_ = 2, *t* = 0.6, and *t*_*a*_ = 0.6 Both lattices are finite in size, containing 8 unit-cells in System 1. An infinite extension of these lattices can be used to verify that they indeed obey Condition 2.

To discriminate or extract the BIC modes from the extended bulk modes, we use a “loss mask” in the bulk of our exemplary lattices, shown as gray shadow region in Figure 4a1,a2. The loss mask here means that we have introduced on-site losses (on-site imaginary propagation constant 0.1*i*) at every lattice site in the gray shaded region. This means that the amplitudes of all modes which overlap with the gray shaded region, i.e., all bulk modes, are decaying (their propagation constants are complex numbers). Moreover, this means that there is a propagation-constant width, equivalent to a resonance width in quantum mechanics, for every bulk mode. The separation between the propagation constants of the bulk mode resonances is smaller than the resonance widths. Moreover, the bulk resonances closest to the zero-energy boundary mode overlap with the zero-energy (shown in Figure 4b1,b2). The boundary zero-energy mode (top red dot) does not decay (no imaginary component) as the loss mask is designed to avoid it.

Next, we test whether the boundary modes will remain robust as BIC modes, so they will not couple to the bulk modes and decay under perturbations. For this purpose, we consider random perturbations in *K* and *H*_*A*_. The perturbed hopping between the *i*th and *j*th waveguides in *H*_*A*_ are of the form *t*_*aij*_ = *t*_*a*_ + δ_*aij*_, where δ_*aij*_ is a perturbation. Similarly, *t*_*i*_ = *t* + δ_*i*_ denotes the *i*th hopping between waveguides in System 1 and 2, i.e., the entries of the coupling matrix *K*. Perturbations δ_*aij*_ and δ_*i*_ are chosen at random from an interval of values [−δ,δ]. Perturbations in *K* satisfy Condition 1 by construction.

In Figure 4c1 we show the overlap of 50 eigenvalue plots in the complex plane calculated for different random perturbations with δ = 0.5 for the lattice structure in Figure 4a1 satisfying Condition 1. It is evident that in Figure 4c1 the eigenvalue of the boundary state (top red dot) remains at zero energy for all perturbations (both real and imaginary components). The same conclusion is found in Figure 4c2 when corresponding perturbation studies are carried out for the multi-BIC lattice shown in Figure 4a2. The representative zero-energy modes corresponding to the systems in Figure 4a1,a2 are plotted in Figure 4d1,d2. The modes are localized at the top-left edge, with characteristic amplitude and phase structure manifesting their topological nature. The persistence of localized modes for a sample of 50 random perturbations (especially in the presence of the loss mask) confirms their robustness as topological BIC modes, with no resonant decay into the bulk whatsoever.^35^

## Conclusion

In this work, we have introduced a practical method for generating topological BICs.^53^ We create a topological BIC system by coupling two subsystems, where one of them is a TI featuring an in-gap boundary state, and the other features a continuous spectrum. We identify the conditions under which the in-gap state is converted into a BIC by coupling the two subsystems. The method is based on the recently developed concept of SubSy;^42^ however, in principle it can be applied even in systems where the SubSy concept is not applicable. We have demonstrated experimentally and proved theoretically the effectiveness of our scheme using the platform of photonic lattices, but the concept may be applied in other platforms such as engineered metasurfaces,^54^ or extended to parity-time symmetric lattices and non-Hermitian systems.^13,55^ The present theory is based on the tight-binding model. We believe that it can be developed further for photonic systems that host BICs but which cannot be described by simple tight-binding models, such as those in refs (57–59) , or in photonic systems involving higher orbitals, such as *p*-orbital HOTIs in ref (60). However, these developments of the theory are beyond the scope of this work. Our discovery can be useful for the creation of intricate topological systems supporting multiple BICs. We believe that this work not only broadens the understanding of topological phenomena in physics but also provides a new pathway for applications in advanced topological device design. In this context, the ability to generate and manipulate topological BICs could bring about important implications for the development of ultrahigh-Q devices and other applications such as BIC lasers.^15,56^

## Supporting Information Available

The Supporting Information contains additional experiments of BIC-mode excitation in a multi-BIC system (PDF)

## References

[ref1] von Neumann J.; Wigner E. Uber merkwürdige diskrete Eigenwerte. Uber das Verhalten von Eigenwerten bei adiabatischen Prozessen. Phys. Z. 1929, 30, 467.

[ref2] Hsu C. W.; Zhen B.; Stone A. D.; Joannopoulos J. D.; Soljačić M. Bound states in the continuum. Nat. Rev. Mater. 2016, 1, 16048 10.1038/natrevmats.2016.48.

[ref3] Sadreev A. F. Interference traps waves in an open system: bound states in the continuum. Rep. Prog. Phys. 2021, 84, 055901 10.1088/1361-6633/abefb9. 33730696

[ref4] Plotnik Y.; Peleg O.; Dreisow F.; Heinrich M.; Nolte S.; Szameit A.; Segev M. Experimental observation of optical bound states in the continuum. Phys. Rev. Lett. 2011, 107, 183901 10.1103/PhysRevLett.107.183901. 22107630

[ref5] Hsu C. W.; Zhen B.; Lee J.; Chua S.-L.; Johnson S. G.; Joannopoulos J. D.; Soljačić M. Observation of trapped light within the radiation continuum. Nature 2013, 499, 188–191. 10.1038/nature12289. 23846657

[ref6] Zhen B.; Hsu C. W.; Lu L.; Stone A. D.; Soljačić M. Topological nature of optical bound states in the continuum. Phys. Rev. Lett. 2014, 113, 257401 10.1103/PhysRevLett.113.257401. 25554906

[ref7] Doeleman H. M.; Monticone F.; den Hollander W.; Alù A.; Koenderink A. F. Experimental observation of a polarization vortex at an optical bound state in the continuum. Nat. Photonics 2018, 12, 397–401. 10.1038/s41566-018-0177-5.

[ref8] Jin J.; Yin X.; Ni L.; Soljačić M.; Zhen B.; Peng C. Topologically enabled ultrahigh-Q guided resonances robust to out-of-plane scattering. Nature 2019, 574, 501–504. 10.1038/s41586-019-1664-7. 31645728

[ref9] Koshelev K.; Bogdanov A.; Kivshar Y. Meta-optics and bound states in the continuum. Sci. Bull. 2019, 64, 836–842. 10.1016/j.scib.2018.12.003. 36659673

[ref10] Marinica D. C.; Borisov A. G.; Shabanov S. V. Bound states in the continuum in photonics. Phys. Rev. Lett. 2008, 100, 183902 10.1103/PhysRevLett.100.183902. 18518374

[ref11] Molina M. I.; Miroshnichenko A. E.; Kivshar Y. S. Surface bound states in the continuum. Phys. Rev. Lett. 2012, 108, 070401 10.1103/PhysRevLett.108.070401. 22401179

[ref12] Yang Y.; Peng C.; Liang Y.; Li Z.; Noda S. Analytical perspective for bound states in the continuum in photonic crystal slabs. Phys. Rev. Lett. 2014, 113, 037401 10.1103/PhysRevLett.113.037401. 25083664

[ref13] Kodigala A.; Lepetit T.; Gu Q.; Bahari B.; Fainman Y.; Kanté B. Lasing action from photonic bound states in continuum. Nature 2017, 541, 196–199. 10.1038/nature20799. 28079064

[ref14] Gomis-Bresco J.; Artigas D.; Torner L. Anisotropy-induced photonic bound states in the continuum. Nat. Photonics 2017, 11, 232–236. 10.1038/nphoton.2017.31.

[ref15] Ha S. T.; Fu Y. H.; Emani N. K.; Pan Z.; Bakker R. M.; Paniagua-Domínguez R.; Kuznetsov A. I. Directional lasing in resonant semiconductor nanoantenna arrays. Nat. Nanotechnol. 2018, 13, 1042–1047. 10.1038/s41565-018-0245-5. 30127475

[ref16] Fan K.; Shadrivov I. V.; Padilla W. J. Dynamic bound states in the continuum. Optica 2019, 6, 169 10.1364/OPTICA.6.000169.

[ref17] Yesilkoy F.; Arvelo E. R.; Jahani Y.; Liu M.; Tittl A.; Cevher V.; Kivshar Y.; Altug H. Ultrasensitive hyperspectral imaging and biodetection enabled by dielectric metasurfaces. Nat. Photonics 2019, 13, 390–396. 10.1038/s41566-019-0394-6.

[ref18] Yu Z.; Xi X.; Ma J.; Tsang H. K.; Zou C.-L.; Sun X. Photonic integrated circuits with bound states in the continuum. Optica 2019, 6, 1342 10.1364/OPTICA.6.001342.

[ref19] Overvig A.; Yu N.; Alù A. Chiral quasi-bound states in the continuum. Phys. Rev. Lett. 2021, 126, 073001 10.1103/PhysRevLett.126.073001. 33666456

[ref20] Hwang M.-S.; Lee H.-C.; Kim K.-H.; Jeong K.-Y.; Kwon S.-H.; Koshelev K.; Kivshar Y.; Park H.-G. Ultralow-threshold laser using super-bound states in the continuum. Nat. Commun. 2021, 12, 4135 10.1038/s41467-021-24502-0. 34226557 PMC8257597

[ref21] Chen Z.; Yin X.; Jin J.; Zheng Z.; Zhang Z.; Wang F.; He L.; Zhen B.; Peng C. Observation of miniaturized bound states in the continuum with ultra-high quality factors. Sci. Bull. 2022, 67, 359–366. 10.1016/j.scib.2021.10.020. 36546087

[ref22] Huang L.; Zhang W.; Zhang X. Moiré quasibound states in the continuum. Phys. Rev. Lett. 2022, 128, 253901 10.1103/PhysRevLett.128.253901. 35802444

[ref23] Chen Y.; Deng H.; Sha X.; Chen W.; Wang R.; Chen Y. H.; Wu D.; Chu J.; Kivshar Y. S.; Xiao S.; et al. Observation of intrinsic chiral bound states in the continuum. Nature 2023, 613, 474–478. 10.1038/s41586-022-05467-6. 36653568

[ref24] Joseph S.; Pandey S.; Sarkar S.; Joseph J. Bound states in the continuum in resonant nanostructures: an overview of engineered materials for tailored applications. Nanophotonics 2021, 10, 4175–4207. 10.1515/nanoph-2021-0387.

[ref25] Parker R. Resonance effects in wake shedding from parallel plates: some experimental observations. J. Sound Vib. 1966, 4, 62–72. 10.1016/0022-460X(66)90154-4.

[ref26] Parker R. Resonance effects in wake shedding from parallel plates: calculation of resonant frequencies. J. Sound Vib. 1967, 5, 330–343. 10.1016/0022-460X(67)90113-7.

[ref27] Hasan M. Z.; Kane C. L. Colloquium: Topological insulators. Rev. Mod. Phys. 2010, 82, 3045–3067. 10.1103/RevModPhys.82.3045.

[ref28] Qi X.-L.; Zhang S.-C. Topological insulators and superconductors. Rev. Mod. Phys. 2011, 83, 1057–1110. 10.1103/RevModPhys.83.1057.

[ref29] Benalcazar W. A.; Bernevig B. A.; Hughes T. L. Quantized electric multipole insulators. Science 2017, 357, 61–66. 10.1126/science.aah6442. 28684520

[ref30] Peterson C. W.; Benalcazar W. A.; Hughes T. L.; Bahl G. A quantized microwave quadrupole insulator with topologically protected corner states. Nature 2018, 555, 346–350. 10.1038/nature25777. 29542690

[ref31] El Hassan A.; Kunst F. K.; Moritz A.; Andler G.; Bergholtz E. J.; Bourennane M. Corner states of light in photonic waveguides. Nat. Photonics 2019, 13, 697–700. 10.1038/s41566-019-0519-y.

[ref32] Roushan P.; Seo J.; Parker C. V.; Hor Y. S.; Hsieh D.; Qian D.; Richardella A.; Hasan M. Z.; Cava R. J.; Yazdani A. Topological surface states protected from backscattering by chiral spin texture. Nature 2009, 460, 1106–1109. 10.1038/nature08308. 19668187

[ref33] Kim S.; Yoshizawa S.; Ishida Y.; Eto K.; Segawa K.; Ando Y.; Shin S.; Komori F. Robust Protection from Backscattering in the Topological Insulator Bi_1.5_Sb_0.5_Te_1.7_Se_1.3_. Phys. Rev. Lett. 2014, 112, 136802 10.1103/PhysRevLett.112.136802. 24745448

[ref34] Xiao Y.-X.; Ma G.; Zhang Z.-Q.; Chan C. T. Topological subspace-induced bound state in the continuum. Phys. Rev. Lett. 2017, 118, 166803 10.1103/PhysRevLett.118.166803. 28474943

[ref35] Benalcazar W. A.; Cerjan A. Bound states in the continuum of higher-order topological insulators. Phys. Rev. B 2020, 101, 161116 10.1103/PhysRevB.101.161116. 33274969

[ref36] Cerjan A.; Jürgensen M.; Benalcazar W. A.; Mukherjee S.; Rechtsman M. C. Observation of a higher-order topological bound state in the continuum. Phys. Rev. Lett. 2020, 125, 213901 10.1103/PhysRevLett.125.213901. 33274969

[ref37] Hu Z.; Bongiovanni D.; Jukić D.; Jajtić E.; Xia S.; Song D.; Xu J.; Morandotti R.; Buljan H.; Chen Z. Nonlinear control of photonic higher-order topological bound states in the continuum. Light: Sci. Appl. 2021, 10, 164 10.1038/s41377-021-00607-5. 34376638 PMC8355333

[ref38] Wang Y.; Xie B. Y.; Lu Y. H.; Chang Y. J.; Wang H. F.; Gao J.; Jiao Z. Q.; Feng Z.; Xu X. Y.; Mei F.; et al. Quantum superposition demonstrated higher-order topological bound states in the continuum. Light: Sci. Appl. 2021, 10, 173 10.1038/s41377-021-00612-8. 34462419 PMC8405621

[ref39] Liu L.; Li T.; Zhang Q.; Xiao M.; Qiu C. Universal mirror-stacking approach for constructing topological bound states in the continuum. Phys. Rev. Lett. 2023, 130, 106301 10.1103/PhysRevLett.130.106301. 36962038

[ref40] Qian L.; Zhang W.; Sun H.; Zhang X. Non-Abelian Topological Bound States in the Continuum. Phys. Rev. Lett. 2024, 132, 046601 10.1103/PhysRevLett.132.046601. 38335357

[ref41] Fu L. Topological crystalline insulators. Phys. Rev. Lett. 2011, 106, 106802 10.1103/PhysRevLett.106.106802. 21469822

[ref42] Wang Z.; Wang X.; Hu Z.; Bongiovanni D.; Jukić D.; Tang L.; Song D.; Morandotti R.; Chen Z.; Buljan H. Sub-symmetry-protected topological states. Nat. Phys. 2023, 19, 992–998. 10.1038/s41567-023-02011-9.

[ref43] Su W. P.; Schrieffer J. R.; Heeger A. J. Solitons in polyacetylene. Phys. Rev. Lett. 1979, 42, 1698–1701. 10.1103/PhysRevLett.42.1698.

[ref44] Su W. P.; Schrieffer J. R.; Heeger A. J. Soliton excitations in polyacetylene. Phys. Rev. B 1980, 22, 2099–2111. 10.1103/PhysRevB.22.2099.

[ref45] Malkova N.; Hromada I.; Wang X.; Bryant G.; Chen Z. Observation of optical Shockley-like surface states in photonic superlattices. Opt. Lett. 2009, 34, 1633 10.1364/OL.34.001633. 19488131

[ref46] Keil R.; Zeuner J. M.; Dreisow F.; Heinrich M.; Tünnermann A.; Nolte S.; Szameit A. The random mass Dirac model and long-range correlations on an integrated optical platform. Nat. Commun. 2013, 4, 1368 10.1038/ncomms2384. 23340408

[ref47] Xiao M.; Zhang Z. Q.; Chan C. T. Surface impedance and bulk band geometric phases in one-dimensional systems. Phys. Rev. X 2014, 4, 021017 10.1103/PhysRevX.4.021017.

[ref48] Kruk S.; Slobozhanyuk A.; Denkova D.; Poddubny A.; Kravchenko I.; Miroshnichenko A.; Neshev D.; Kivshar Y. Edge states and topological phase transitions in chains of dielectric nanoparticles. Small 2017, 13, 1603190 10.1002/smll.201603190. 28079975

[ref49] Poddubny A.; Miroshnichenko A.; Slobozhanyuk A.; Kivshar Y. Topological Majorana states in zigzag chains of plasmonic nanoparticles. ACS Photonics 2014, 1, 101–105. 10.1021/ph4000949.

[ref50] Blanco-Redondo A.; Bell B.; Oren D.; Eggleton B. J.; Segev M. Topological protection of biphoton states. Science 2018, 362, 568–571. 10.1126/science.aau4296. 30385574

[ref51] Weimann S.; Kremer M.; Plotnik Y.; Lumer Y.; Nolte S.; Makris K. G.; Segev M.; Rechtsman M. C.; Szameit A. Topologically protected bound states in photonic parity–time-symmetric crystals. Nat. Mater. 2017, 16, 433–438. 10.1038/nmat4811. 27918567

[ref52] Xia S.; Kaltsas D.; Song D.; Komis I.; Xu J.; Szameit A.; Buljan H.; Makris K. G.; Chen Z. Nonlinear tuning of PT symmetry and non-Hermitian topological states. Science 2021, 372, 72–76. 10.1126/science.abf6873. 33795453

[ref53] Wang X.; Topological bound states in the continuum without global symmetry. In Nonlinear Opt.; Optica Publishing Group, 2023; p W2A. 2.

[ref54] Regensburger A.; Miri M.-A.; Bersch C.; Näger J.; Onishchukov G.; Christodoulides D. N.; Peschel U. Observation of defect states in PT-symmetric optical lattices. Phys. Rev. Lett. 2013, 110, 223902 10.1103/PhysRevLett.110.223902. 23767725

[ref55] Rivero J. D.; Fleming C.; Qi B.; Feng L.; Ge L. Robust zero modes in non-Hermitian systems without global symmetries. Phys. Rev. Lett. 2023, 131, 223801 10.1103/PhysRevLett.131.223801. 38101337

[ref56] Ren Y.; Li P.; Liu Z.; Chen Z.; Chen Y.-L.; Peng C.; Liu J. Low-threshold nanolasers based on miniaturized bound states in the continuum. Sci. Adv. 2022, 8, eade8817 10.1126/sciadv.ade8817. 36563161 PMC9788758

[ref57] Pocock S. R.; Xiao X.; Huidobro P. A.; Giannini V. Topological plasmonic chain with retardation and radiative effects. ACS Photonics 2018, 5, 2271–2279. 10.1021/acsphotonics.8b00117.

[ref58] Ardizzone V.; Riminucci F.; Zanotti S.; Gianfrate A.; Efthymiou-Tsironi M.; Suàrez-Forero D. G.; Todisco F.; De Giorgi M.; Trypogeorgos D.; Gigli G.; et al. Polariton Bose–Einstein condensate from a bound state in the continuum. Nature 2022, 605, 447–452. 10.1038/s41586-022-04583-7. 35585343

[ref59] Heilmann R.; Salerno G.; Cuerda J.; Hakala T. K.; Törmä P. Quasi-BIC mode lasing in a quadrumer plasmonic lattice. ACS Photonics 2022, 9, 224–232. 10.1021/acsphotonics.1c01416. 35083367 PMC8780794

[ref60] Zhang Y.; Bongiovanni D.; Wang Z.; Wang X.; Xia S.; Hu Z.; Song D.; Jukić D.; Xu J.; Morandotti R.; Buljan H.; Chen Z. Realization of photonic p-orbital higher-order topological insulators. eLight 2023, 10.1186/s43593-022-00039-7.

