# Supplementary material for: Construction of Topological Bound States in the Continuum Via Subsymmetry
Source: ACS Photonics. 2024 Jul 17;11(8):3213–20. doi: 10.1021/acsphotonics.4c00600 (PMC11342891; doi:10.1021/acsphotonics.4c00600)
Supplement: Supplementary file 1 — ph4c00600_si_001.pdf [file ph4c00600_si_001.pdf]

Supporting Information

Construction of topological bound states in the continuum via sub-symmetry

Xiangdong Wang[1†], Domenico Bongiovanni[1,2†], Ziteng Wang[1†], Amgad Abdrabou[3], Zhichan Hu[1], Dario Jukić[4], Daohong Song[1,5], Roberto Morandotti[2], Ramy El-Ganainy[6], Zhigang Chen[1,5*], and Hrvoje Buljan[1,7*]

[1]*TEDA Applied Physics Institute and School of Physics, Nankai University, Tianjin 300457, China*
[2]*INRS-EMT, 1650 Blvd. Lionel-Boulet, Varennes, Quebec J3X 1S2, Canada*
[3]*Elmore Family School of Electrical and Computer Engineering, Purdue University, West Lafayette, IN 47907, USA*
[4]*Faculty of Civil Engineering, University of Zagreb, A. Kačića Miošića 26, 10000 Zagreb, Croatia*
[5]*Collaborative Innovation Center of Extreme Optics, Shanxi University, Taiyuan, Shanxi 030006, China*
[6]*Department of Physics, Michigan Technological University, Houghton, Michigan 49931, USA*
[7]*Department of Physics, Faculty of Science, University of Zagreb, Bijenička c. 32, Zagreb 10000, Croatia*
[†]*These authors contributed equally to this work*
**zgchen@nankai.edu.cn, *hbuljan@phy.hr*

Experimental results with structured BIC mode excitation

In the main text, in experiments presented in Fig. 1c1 and Figs. 3a1,a2, we have excited one boundary lattice site, which excites dominantly the pertinent BIC mode, but somewhat also unwanted bulk modes. To excite the boundary BIC mode more precisely, we need to tailor the initial excitation. For example, to excite the top left corner mode in the structure of Fig. 3a1 of the main text, we need to excite the first and the third lattice site in the upper row from the left, and arrange the phases such that the light in the third waveguide is out-of-phase with respect to that in the first waveguide (theoretically, there is a negligible amount of mode amplitude in the 5th waveguide, 7th waveguide and so on but we can safely neglect these amplitudes for the parameters used in our experiment). This type of structured beam excitation is illustrated in Figs. S1a1 (intensity structure) and a3 (phase structure). Evolution from such an initial condition is presented in Figs. S1b1 (experiment) and c1 (simulation). Evidently, we have dominantly excited the BIC mode as there is no unwanted spreading dynamics in the intensity.

In contrast, when we excite the same waveguides with identical intensity (Fig. S1a2) but different phase structure (Fig. S1a4, which shows in-phase-excitation), in the subsequent propagation we clearly see coupling of light into the neighboring B sublattice sites.

We performed equivalent experiments and found equivalent conclusions for another BIC mode in the same lattice structure (when we excite the BIC mode in the vertically middle row). In Figs. S1b3 and c3, we show experiments and simulations corresponding to this mode excitation (out-of-phase initial condition), and in Figs. S1b4, c4 we show evolution from the in-phase initial condition for a direct comparison. These types of experimental results underpin the conclusions from the main text that we have experimentally

demonstrated the topological BIC modes.

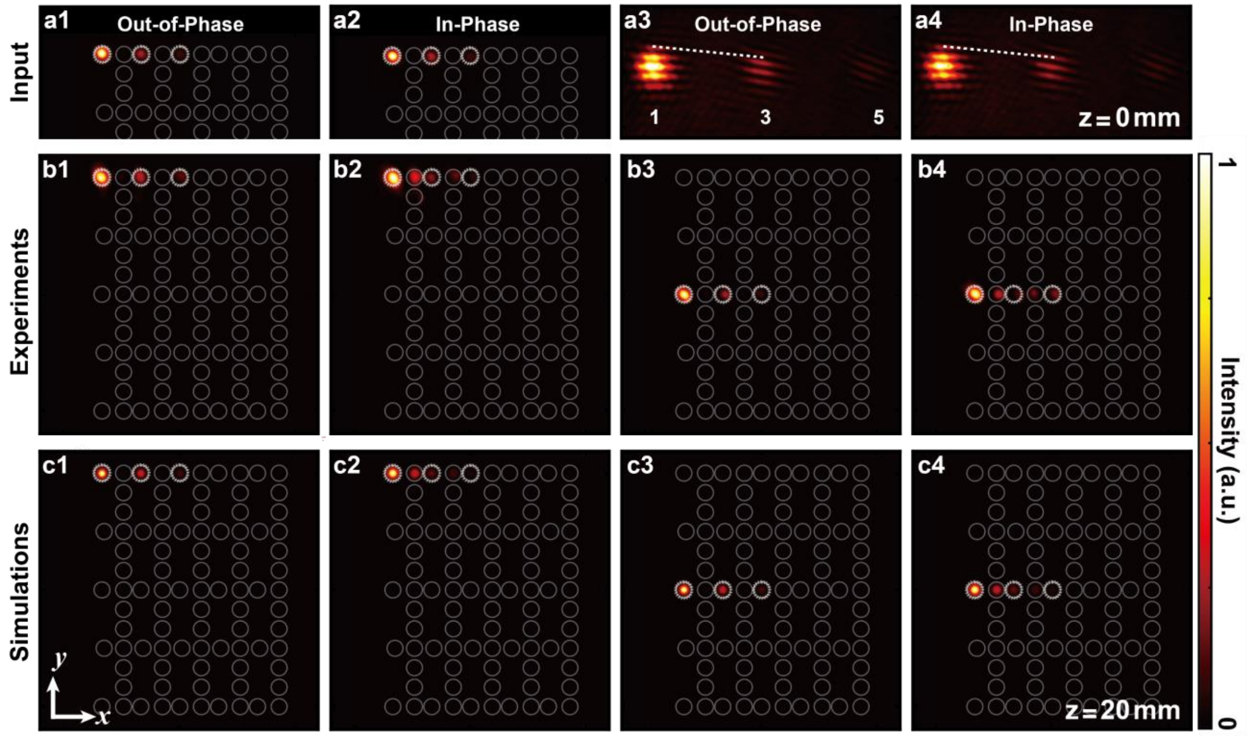

Figure S1. Experimental and numerical results of topologically nontrivial mode excitation in a multi-BIC system. (a1,a2) Input intensity distributions of out-of-phase (a1) and in-phase (a2) initial conditions; the input intensity distribution matches the BIC mode distribution. (a3, a4) The interference patterns (enlarged for a clearer visibility) resulting from the interference of the input beams in (a1) and (a2) with a plane wave. (b1-b4) Output intensity measurements after 20-mm propagation of the probe beams through the multi-BIC lattice. (b1,b3) Intensity patterns arising from the out-of-phase initial condition for the two BIC modes present in this structure; the initial and final intensity patterns coincide. (b2,b4) The same as (b1,b3) for the in-phase initial condition; the final intensity has spread into the neighboring sites. (c1-c4) Numerical simulations corresponding to experimental observations shown in the panels (b1-b4).
